# Management of Psychosis Associated with Graves' Disease: A Rare Case Report

**DOI:** 10.1155/2022/4685870

**Published:** 2022-12-29

**Authors:** Ozge Ceren Amuk Williams, Mouad Abdulrahim, Victoria Davis, Charles Jenson, Ayush Anand, Anil Krishna Bachu

**Affiliations:** ^1^Griffin Memorial Hospital, Norman, USA; ^2^Baptist Health, Little Rock, USA; ^3^B.P. Koirala Institute of Health Sciences, Dharan, Nepal

## Abstract

Graves' disease is an autoimmune disease in which patients can rarely present with psychiatric symptoms. In these patients, detailed history with psychiatric evaluation using a mental status examination is crucial for the early identification of psychiatric manifestations. Early intervention with medical and surgical therapy can help effectively treat the condition and prevent adverse outcomes such as catatonia. We reported the case of a 25-year-old African American female with Graves' disease who had significant stressors and presented with auditory hallucinations. She was diagnosed with psychosis secondary to Graves' disease and was managed medically using antithyroid drugs and beta-blockers. On failure of medical therapy, a surgical approach was employed. The patient was managed successfully, and her condition improved. Our case highlights that the importance of early intervention in these cases can lead to successful outcomes in patients with Graves' disease-induced psychosis.

## 1. Introduction

Graves' disease is an autoimmune disease and the most common cause of hyperthyroidism, and it is more common in women, smokers, and patients with autoimmune disorders [1, 2]. Stressful life events may also be a risk factor for Graves' disease [3]. These patients usually present with acute onset with peculiar symptoms of hyperthyroidism, such as heat intolerance, weight loss, palpitation, irritability, disturbed sleep, and menstrual irregularity [1, 2, 4, 5]. The diagnosis of Graves' disease can be made based on clinical evaluation, laboratory investigations, and imaging studies [1, 4]. Laboratory studies include serum T3, T4, and thyroid autoantibodies [1]. Though thyroid ultrasonography is sufficient for diagnosis, other imaging modalities such as radio-iodide uptake and computed tomography scan of the neck may be needed [1]. Medical therapy involves using beta-blockers, antithyroid medications, and radioactive iodine [4]. Moreover, thyroidectomy may be required if the patients do not respond well to medical therapy [1].

Graves' disease can also have psychiatric manifestations such as anxiety, depression, delirium, toxic psychosis, and bipolar disorders [5]. With an incidence of about 1%, psychosis is one of the rare presentations of Graves' disease [6]. A detailed history and psychiatric evaluation are required to diagnose these cases and assess the disease's severity. Often, antipsychotic drugs and adequate management of thyroid disorders improve the patient's condition [6]. Herein, we present the case of a 25-year-old African female, a known case of Graves' disease presented with psychotic symptoms. On failure of initial medical therapy, a surgical approach was employed, and the patient was managed successfully.

## 2. Case Report

A 25-year-old African American female, a known case of Graves' disease for three years, presented with auditory hallucinations for three months. Initially, she had occasional auditory hallucinations of a woman screaming for help. Over the past three months, the auditory hallucinations became more frequent and distressing enough to interfere with her sleep. In addition, she had difficulty falling asleep, anhedonia, decreased attention and concentration, lack of energy, and suicidal ideation. Her stressors included a recent history of sexual assault and the loss of her cousin due to gun violence. The patient had multiple admissions with symptoms of thyrotoxicosis (heat intolerance, palpitations, and headache with undetectable TSH due to noncompliance with medications) and a history of atrial fibrillation.

**Figure 1:**
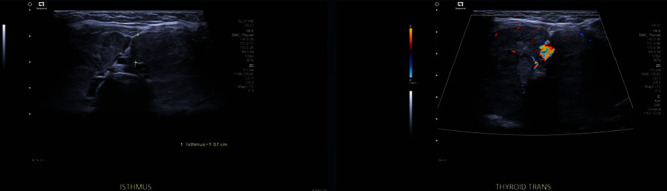
Ultrasound scan of the thyroid shows a markedly enlarged diffusely heterogenous thyroid gland without well-defined nodules.

On examination, she was hemodynamically stable, and her systemic examination was normal. On the mental status examination, the patients appeared to be engaging with hallucinations, reduced eye contact, disengaged facial expression, and restlessness. Her speech was slow, minimal, monotonous, and decreased volume. She had a low mood and blunted affect. Her lab investigations showed (Table 1) anemia and thyroid-stimulating hormone (TSH) of less than 0.008. An ultrasound scan of the thyroid revealed diffuse goiter (Figure 1).

Based on her clinical evaluation, a diagnosis of Graves' disease-induced psychosis was made. Her psychotic symptoms were managed with Tab Quetiapine 50 mg PO OD for two days and 100 mg PO in two divided doses for one day. For depressive symptoms, Tab Sertraline 200 mg PO QPM for two days was given. Quetiapine was gradually increased to 400 mg daily over five days. Also, Sodium valproate was added to augment depression treatment. Trazodone was added at night to help the patient sleep, and Sertraline was discontinued. The medical management of thyrotoxicosis was done with Tab Methimazole PO OD 10 mg and Tab Propranolol 10 mg PO BID. Due to worsening thyrotoxicosis, a total thyroidectomy was done on the 7^th^ day. Over the course of the hospital stay, the frequency of auditory hallucinations decreased gradually to one per day, and her mood improved significantly. The patient was discharged with Tab Quetiapine 400 mg QPM for her psychiatric symptoms. Also, Levothyroxine 150 mcg and Propranolol 10 mg BID were given, and she was advised to follow up after one month in OPD.

## 3. Discussion

Graves' disease is more common among women, and stressful life events can be a risk factor [2, 3]. Similar to this, the patient was a female with significant stressors, including sexual assault and the death of a close relative. We considered a differential diagnosis of major depressive disorder with psychosis. Since the patient had a long history of Graves' disease and was noncompliant with medications, the diagnosis of thyrotoxicosis-induced psychosis was made.

Iskandar et al. reported that untreated hyperthyroidism might lead to paranoia, altered sleep, and catatonia [7]. The psychosis can be managed with the administration of antipsychotic agents [8]. However, caution should be maintained as treatment with antipsychotics may lead to catatonia [9]. In addition, mood stabilizers and antidepressant can be used for better control of symptoms [8, 10, 11]. Also, adequate management of thyrotoxicosis using antithyroid drugs, anxiolytics, and electroconvulsive therapy is crucial for improving medical and psychotic symptoms [6, 12]. Bennett et al. reported successful management of psychosis without antipsychotics in a female patient using Carbimazole and beta-blockers [13]. Also, Asif et al. reported successful management of psychosis due to Graves' disease using Methimazole, Propranolol, and Hydrocortisone [14]. Another study reported the remission of psychotic symptoms in four weeks of Methimazole use in a patient with psychosis due to Graves' disease [15]. However, a surgical approach is warranted if the medical management fails to control thyrotoxicosis [1]. Marian et al. reported a case of a female in her thirties who had thyrotoxicosis successfully managed with thyroidectomy after the medical therapy failed [16]. In our case, the psychosis was managed with antipsychotics, antidepressants, and mood stabilizers. Methimazole, a thyroid hormone inhibitor, was used to treat thyrotoxicosis. Since the medical management failed, the patient was managed surgically by total thyroidectomy. The prompt management helped prevent catatonia, and the patient's condition improved.

## 4. Conclusion

We showed the case of successful management of psychosis induced by thyrotoxicosis in a female patient with Graves' disease. Our case highlighted that psychosis symptoms could be successfully managed by a combination of antipsychotic drugs and adequate management of underlying thyrotoxicosis. Also, prompt management can successfully prevent catatonia. If the medical management fails, a surgical approach should be employed to control thyrotoxicosis. Since the presentation is rare, a high index of clinical suspicion is required to identify and manage these cases. More studies are required to understand the pathophysiology behind psychosis due to Graves' disease.

## Figures and Tables

**Table 1:** Laboratory investigations of the patient.

Investigations	Result	Reference range
Hb (g/dl)	11.6	12.0-16.0
TLC (K/mm^3^)	10.8	5.0-10.0
RBC (million/mm^3^)	4.68	4.10-5.00
PCV (%)	35.9	36.0-48.0
MCV (fl)	77	80-100
MCH (pg)	25	27.0-31.0
MCHC (g/dl)	32	32.0-36.0
MPV (fl)	10.7	7.5-12.5
Serum sodium (meq/l)	137	136-145
Serum potassium (meq/l)	4.3	3.5-5.1
Serum calcium (mg/dl)	8.5	8.4-10.2
ALT (IU/l)	20	5-34
AST (IU/l)	12	0-55
GGT (IU/l)	40	12-43
TGL (mg/dl)	91	0-150
HDL-C (mg/dl)	34	40-60
LDL-C (mg/dl)	85	0-99
TSH (*μ*IU/ml)	<0.008	0.35-4.94
FT4 (*μ*g/dl)	4.44	1.65-4.07
T4 total (*μ*g/dl)	11.6	4.87-11.72
T3 uptake (%)	38.3	15-50%

Hb = hemoglobin; TLC = total leukocyte count; RBC = red blood cell; PCV = packed cell volume; MCV = mean corpuscular volume; MCH = mean corpuscular hemoglobin; MCHC = mean corpuscular hemoglobin concentration; ALT = alanine transaminase; AST = aspartate transaminase; GGT = gamma-glutamyl transferase; TGL = triglyceride; HDL-C = high-density lipoprotein; LDL = low-density lipoprotein; TSH = thyroid-stimulating hormone.

## Data Availability

All relevant data related to this case is available within this manuscript.

## Additional Points

*Guarantor*. AKB is the guarantor of this manuscript.
